# Enhanced Effects of Riboflavin/UVA Crosslinking, N-Acetylcysteine, and Polydatin on Collagenase Resistance in Human Corneal and Scleral Tissue: A Pilot Ex Vivo Study

**DOI:** 10.3390/jcm15207836

**Published:** 2026-10-11

**Authors:** Cosimo Mazzotta, Francesco Cappellani, Mayank A. Nanavaty, Marco Zeppieri, Alessandro Avitabile, Alessandro Meduri, Giovanni Scapagnini

**Affiliations:** 1 Department of Medicine and Surgery, University Kore of Enna, 94100 Enna, Italy; 2 Siena Crosslinking Center, Monteriggioni, 53035 Siena, Italy; 3 University Hospitals Sussex NHS Foundation Trust, Brighton BN2 5BE, UK; 4 Department of Ophthalmology, University Hospital of Udine, 33100 Udine, Italy; 5 Department of Medicine, Surgery and Health Sciences, University of Trieste, 34129 Trieste, Italy; 6 Neurovisual Science Technology (NEST), 95123 Catania, Italy; 7 Ophthalmology Clinic, Department of Biomedical and Dental Sciences and Morphofunctional Imaging, University of Messina, 98122 Messina, Italy; 8 Department of Medicine and Health Sciences “V. Tiberio”, University of Molise, 86100 Campobasso, Italy; g.scapagnini@gmail.com

**Keywords:** corneal collagen crosslinking, scleral crosslinking, keratoconus, progressive myopia, collagenase, N-acetylcysteine, polydatin

## Abstract

**Purpose**: To investigate whether N-acetylcysteine (NAC) and *Polygonum cuspidatum* extract enhance riboflavin/ultraviolet-A crosslinking (CXL), exploring a potential adjunctive strategy for corneal and scleral tissue stabilization. **Methods**: In this pilot ex vivo study, 12 human corneal buttons and 12 scleral strips were assigned to six groups: untreated controls, CXL alone, adjunctive treatment alone, and adjunctive treatment before, after, or both before and after CXL. Accelerated CXL was performed at 9 mW/cm^2^ for 10 min (5.4 J/cm^2^). Resistance to collagenase-mediated degradation and corneal protein release were evaluated. **Results**: CXL increased corneal degradation time from 7 ± 2 to 14 ± 2 days and scleral degradation time from 10 ± 2 to 19 ± 3 days. Combined pre- and post-CXL adjunctive treatment further prolonged resistance to 21 ± 2 days in corneas (*p* < 0.001) and 27 ± 3 days in sclerae (*p* < 0.01), compared with CXL alone. Corneal protein release decreased by 84% and 52% at days 5 and 10, respectively (both *p* < 0.01). **Conclusions**: These preliminary findings suggest that biochemical adjuncts may enhance CXL-mediated extracellular matrix stabilization, opening new perspectives for ocular wall reinforcement. This approach could support future therapeutic strategies targeting corneal weakening in keratoconus and scleral remodeling in progressive myopia, with potential extensions to refractive surgery in thin corneas. Further experimental and clinical validation is warranted.

## 1. Introduction

Corneal collagen crosslinking (CXL) using riboflavin and ultraviolet-A (UVA) irradiation has become an established treatment for progressive keratoconus and corneal ectatic disorders [1]. By inducing the formation of reactive oxygen species, riboflavin/UVA irradiation promotes the development of additional covalent bonds between stromal collagen fibers, resulting in increased biomechanical rigidity and stabilization of the corneal extracellular matrix [2,3]. Clinical studies have consistently demonstrated the efficacy of CXL in slowing or halting ectatic progression and preserving visual function in keratoconus (KC) patients [4]. Beyond its well-established biomechanical effects, riboflavin/UVA CXL also induces relevant biochemical changes within the corneal extracellular matrix [4]. Photo-oxidative crosslink formation modifies the molecular interactions within the stromal collagen matrix and increases its resistance to proteolytic degradation. In particular, Spoerl et al. demonstrated that riboflavin/UVA crosslinking significantly increases the resistance of corneal collagen to enzymatic digestion by pepsin, trypsin, and collagenase [5]. This enhanced resistance to enzymatic degradation may contribute to extracellular matrix stabilization, particularly in conditions characterized by increased proteolytic activity and progressive stromal weakening.

Increased activity of matrix metalloproteinases, collagenases and extracellular matrix degradation are known to contribute to progressive stromal weakening in keratoconus and corneal melting disorders [5]. Growing attention has recently been directed toward adjunctive biochemical strategies capable of modulating extracellular matrix degradation and oxidative stress pathways. N-acetylcysteine (NAC), a thiol-containing antioxidant and precursor of glutathione, has demonstrated anti-inflammatory and antiproteolytic properties, including modulation of matrix metalloproteinase activity [6,7,8].

Similarly, Polydatin, a natural glucoside precursor of resveratrol extracted from *Polygonum cuspidatum* (Pc), exhibits antioxidant and extracellular matrix-protective effects in several experimental models [9]. Such compounds may therefore enhance tissue resistance to enzymatic degradation and may exert complementary anti-proteolytic effects in association with crosslinking [10]. Although the clinical role of corneal crosslinking is now well established in KC and other corneal ectasia, analogous extracellular matrix stabilization strategies may also have potential relevance in scleral remodeling associated with progressive myopia [11,12]. Progressive scleral remodeling, collagen disorganization, scleral thinning and weakening of the scleral collagen-extracellular matrix (ECM) complex play a central role in myopia progression [13,14]. Progressive myopia is increasingly recognized as a disease characterized by collagen disorganization, protease activation, and biomechanical weakening of the scleral ECM, ultimately contributing to equatorial stretching, ocular expansion and axial elongation [15]. Experimental studies [16,17] have demonstrated the feasibility of scleral crosslinking and suggest that increasing scleral rigidity may potentially counteract progressive deformation of the ocular wall [18,19,20]. In human cadaver eyes, scleral collagen crosslinking by ultraviolet activation of riboflavin differentially increases scleral Young’s modulus (YM), with a greater effect in the equatorial than in the posterior sclera and the greatest stiffening observed in the lateral equatorial regions [21]. Crosslinking might be used to arrest progressive myopia or to prevent staphyloma formation [21,22].

The purpose of the present study was therefore to evaluate the resistance of human corneal buttons and scleral strips to collagenase-mediated enzymatic degradation [5] following Riboflavin/UVA CXL, either alone or combined with adjunctive NAC and Pc treatment. By investigating both corneal and scleral tissue stabilization within the same experimental model, this study aims to provide further insight into combined biomechanical and biochemical ECM stabilization strategies with potential translational implications for corneal ectatic disorders and progressive myopia.

## 2. Materials and Methods

### 2.1. Setting and Donor Tissues

This experimental laboratory study was conceived at the Siena International Crosslinking Center, Monteriggioni, Siena, Italy and conducted at the Biotecnica Associates Laboratories, Castelfidardo, Ancona, Italy, in collaboration with the Veneto Eye Bank Foundation, Venice, Italy and the University of Enna Kore Ophthalmology Department. The study was authorized by the Institutional Review Board of the Siena Crosslinking Center (Sclero-Corneal Enzymatic Digestion Study 1, Code: SCED-S1) under the tenets of the Helsinki Declaration and respecting good laboratory practice (GLP) rules.

Twenty-four human donor tissue specimens unsuitable for transplantation, comprising 12 corneal specimens and 12 scleral specimens obtained from 24 different donors, were provided by the Veneto Eye Bank Foundation, Venice, Italy, for experimental research purposes. Samples were stored at 5 ± 3 °C in storage solution (Optisol™, Bausch & Lomb Inc., Rochester, NY, USA) until the beginning of the study.

Twelve corneal buttons were punched using a 6-mm trephine to obtain homogeneous samples. The included corneal buttons measured 6 mm in diameter, with an average central corneal thickness of 503 ± 27 µm. Central thickness was measured by portable ultrasound pachymetry (Pachette 4™, DGH Technology, Inc., Exton, PA, USA).

Similarly, twelve scleral strips measuring 6 mm in length × 2 mm in width were prepared for the study and subsequently allocated to the same six experimental groups, with two specimens per group.

### 2.2. Experimental Groups

Corneal buttons and scleral strips were divided into six experimental groups:

- Group A: untreated controls;
- Group B: riboflavin/UVA CXL alone;
- Group C: NAC/Pc incubation alone;
- Group D: NAC/Pc incubation before CXL;
- Group E: NAC/Pc incubation after CXL;
- Group F: NAC/Pc incubation before and after CXL.

Each of the six experimental groups included two corneal specimens and two scleral specimens (n = 2 per tissue type per group). All specimens represented independent biological samples obtained from different donors; corneal and scleral specimens were not donor-matched.

The complete experimental protocol was independently repeated in a second experimental session using tissues from different donors and the same treatment groups and experimental conditions. The replicate experiment reproduced the same overall pattern of treatment effects observed in the primary experiment. However, because the specimens available for the second session were smaller than those used in the primary experiment, data from the two experimental sessions were not pooled for quantitative statistical analysis, in order to avoid introducing specimen-size heterogeneity as an additional source of variability. Accordingly, the quantitative analyses reported in the present study refer to the standardized specimens from the primary experimental session.

### 2.3. Crosslinking Procedure

Crosslinking (CXL) treatment consisted of 15 min of Riboflavin soaking followed by continuous UVA irradiation for 10 min at 9 mW/cm^2^, corresponding to a total Fluence of 5.4 J/cm^2^. Isotonic dextran-free 0.1% Riboflavin solution was applied during UV-A irradiation every two minutes [23].

### 2.4. NAC/Pc Solution

The adjunctive bioactive solution was prepared by dissolving a 3.7 g mixture containing 400 mg of N-acetylcysteine (NAC) and 200 mg of *Polygonum cuspidatum* (Pc) dry extract in 250 mL of deionized water. Samples assigned to the adjunctive treatment groups were incubated in this solution for 20 min before CXL, 20 min after CXL, or both before and after CXL, according to group allocation.

The formulation provided a final NAC concentration of 1.6 mg/mL (0.16%; approximately 9.8 mM), together with 200 mg of *Polygonum cuspidatum* dry extract in 250 mL of solution. The selected formulation was considered biologically plausible on the basis of previous experimental evidence supporting the antiproteolytic properties of its components. NAC has been shown to inhibit purified corneal collagenase, with an IC_50_ of 2.7 mM against a synthetic substrate and <10 mM when native type I collagen was used as substrate [24]. Moreover, NAC concentrations ranging from 0 to 20 mM have been investigated in human corneal epithelial cells, demonstrating concentration-dependent biological effects and reduced MMP-9 secretion at higher concentrations [6]. Additional ex vivo evidence has demonstrated inhibition of collagenase-mediated corneal degradation by NAC [25]. Experimental studies have also demonstrated marked anti-collagenase activity of *Polygonum cuspidatum* extracts and Polydatin, one of its major stilbene derivatives [10].

The concentrations used in the present pilot study were not intended to represent an optimized dose; rather, a fixed formulation and incubation time were maintained across treatment groups to investigate the effect of adjunctive treatment and its timing relative to CXL.

### 2.5. Enzymatic Degradation Assay

The collagenase-mediated degradation assay was based on the experimental approach originally described by Spoerl et al. [5], with the experimental conditions specified below. Following the assigned treatments, each corneal button (6 mm diameter) or scleral strip (6 × 2 mm) was individually placed in a sterile multi-well plate containing 2 mL of collagenase solution. The enzymatic digestion solution consisted of 0.1% bacterial collagenase A (EC 3.4.24.3, *Clostridium histolyticum*; Roche, Mannheim, Germany), corresponding to 0.1 U/mL, prepared in phosphate-buffered saline (PBS) at pH 7.5.

Each standardized tissue specimen was therefore exposed individually to a fixed 2-mL volume of collagenase solution under identical experimental conditions. Samples were maintained at 25 ± 2 °C and 60 ± 5% relative humidity and evaluated daily until complete tissue degradation. Corneal button diameter and scleral strip length were monitored throughout incubation to assess degradation kinetics. Protein released into the incubation medium from corneal samples was quantified using the Bradford assay [26].

### 2.6. Statistical Analysis

Statistical analyses were performed using GraphPad Prism 9.0 (GraphPad Software, San Diego, CA, USA). Data are presented as means ± standard deviations (SDs). Differences in degradation time among the six experimental groups were assessed using one-way analysis of variance (ANOVA), followed by Tukey’s multiple-comparison post hoc test. For the protein-release kinetics assay, statistical comparisons were restricted to two predefined representative time points, days 5 and 10, selected to assess the early and intermediate phases of collagenase-mediated tissue degradation while avoiding multiple comparisons at every sampling time point. At each of these predefined time points, protein concentrations in the NAC/Polydatin pre/post-CXL group were compared with those in the untreated control group using an unpaired *t*-test. Statistical significance was set at *p* < 0.05.

## 3. Results

**Figure 1:**
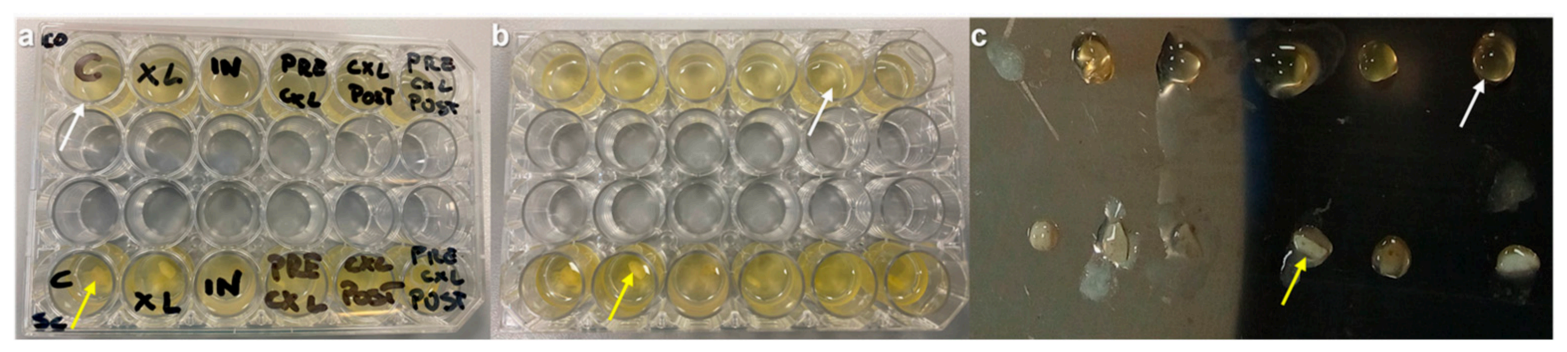
Representative macroscopic appearance of human corneal buttons and scleral strips during collagenase-mediated degradation following riboflavin/UVA crosslinking and adjunctive NAC/Polydatin treatment. (**a**) Experimental groups at the beginning of incubation. White arrows indicate corneal buttons and yellow arrows indicate scleral strips. The six treatment groups were as follows: untreated control; CXL alone (9 mW/cm^2^; 5.4 J/cm^2^); NAC/Polydatin incubation alone; NAC/Polydatin incubation before CXL; NAC/Polydatin incubation after CXL; and NAC/Polydatin incubation both before and after CXL. (**b**) Intermediate phase of enzymatic degradation, showing delayed dissolution of corneal and scleral tissues following CXL, particularly in specimens receiving adjunctive NAC/Polydatin treatment. (**c**) Late phase of incubation, demonstrating greater persistence of structurally preserved tissue in the NAC/Polydatin pre/post-CXL group, whereas untreated specimens showed advanced degradation and dissolution. These representative macroscopic observations are consistent with the quantitative degradation-time and protein-release analyses. CXL = collagen crosslinking; NAC = N-acetylcysteine; Pc = *Polygonum cuspidatum* dry extract.

Representative macroscopic changes observed during collagenase-mediated degradation across the experimental groups are shown in Figure 1.

### 3.1. Corneal Enzymatic Degradation

**Figure 2:**
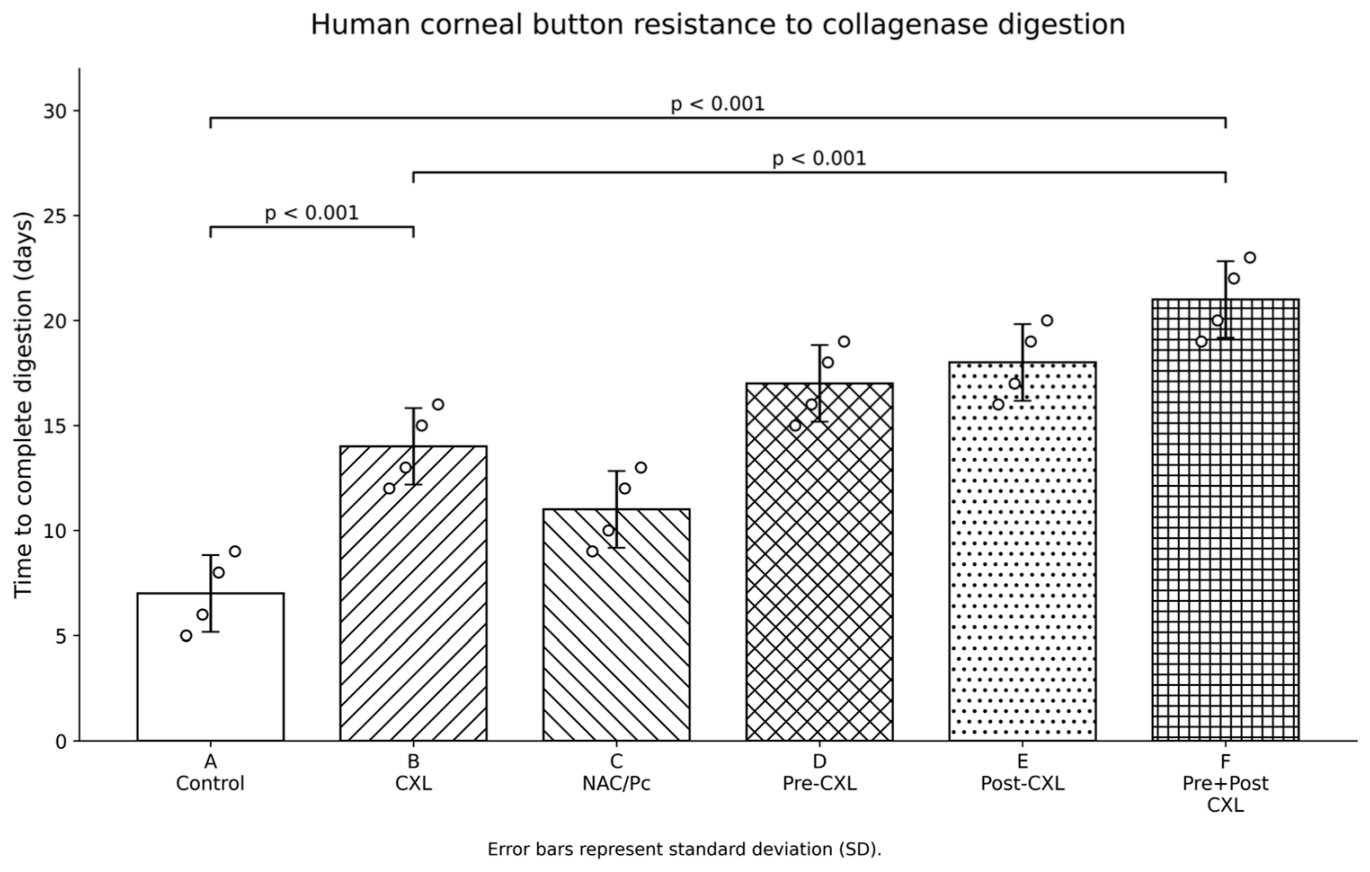
Resistance of human corneal buttons to collagenase-mediated degradation following riboflavin/UVA crosslinking and adjunctive NAC/Polydatin treatment. Mean time to complete collagenase-mediated degradation of human corneal buttons in the six experimental groups. Riboflavin/UVA collagen crosslinking significantly prolonged corneal preservation compared with untreated controls. NAC/Polydatin incubation alone produced only a modest increase in degradation time, whereas combined NAC/Polydatin treatment before and after CXL produced the greatest anti-degradative effect. Individual sample values are displayed, and error bars represent standard deviation (SD). Statistical significance was assessed by one-way ANOVA followed by Tukey’s multiple-comparison post hoc test. The brackets indicate the statistically significant comparisons displayed in the figure: untreated control versus CXL, CXL versus NAC/Polydatin pre/post-CXL, and untreated control versus NAC/Polydatin pre/post-CXL (all *p* < 0.001). CXL = collagen crosslinking; NAC = N-acetylcysteine; Pc = *Polygonum cuspidatum* (Polydatin) dry extract.

**Figure 3:**
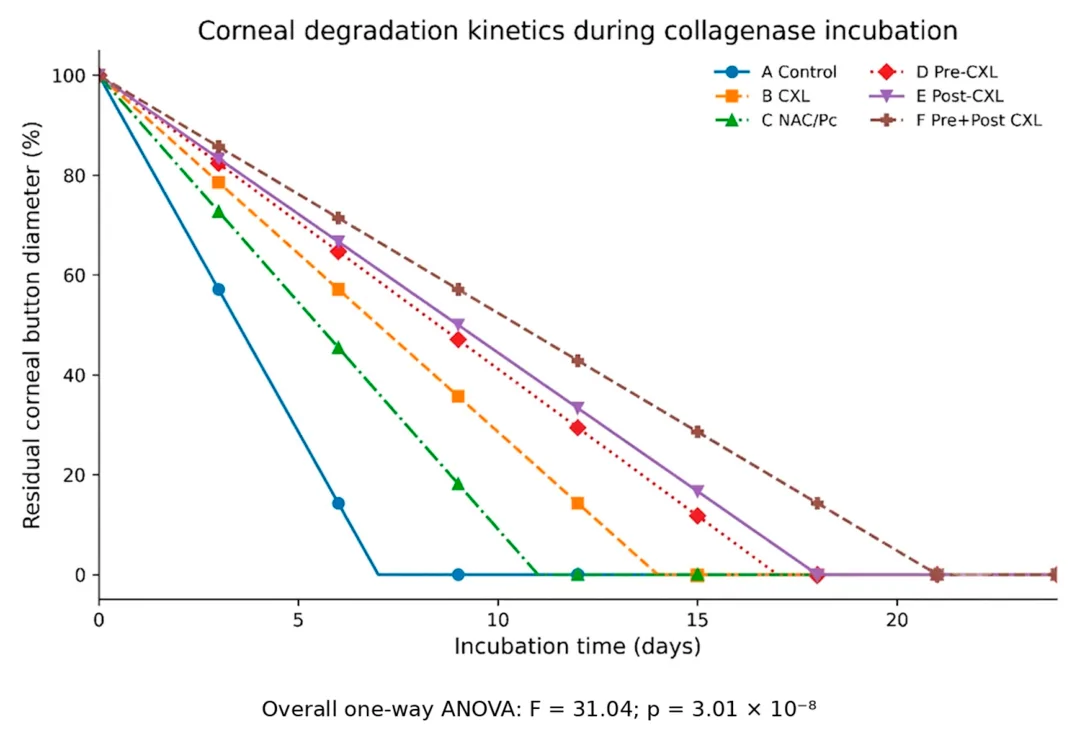
Kinetics of collagenase-mediated degradation of human corneal buttons. Residual corneal diameter (%) was monitored over time in the six experimental groups. Untreated controls showed the fastest degradation, whereas riboflavin/UVA CXL delayed tissue digestion. The addition of NAC/Polydatin further enhanced tissue resistance, with the greatest preservation observed in the NAC/Polydatin pre/post-CXL group. Data are presented as means ± SDs. The overall statistical significance reported in the figure refers to the comparison of complete degradation times among the six experimental groups by one-way ANOVA (F = 31.04, *p* = 3.01 × 10^−8^), followed by Tukey’s multiple-comparison post hoc test; no point-by-point statistical comparisons were performed along the kinetic curves. CXL = collagen crosslinking; NAC = N-acetylcysteine; Pc = *Polygonum cuspidatum* (Polydatin) dry extract.

Untreated corneal buttons (Group A) underwent complete collagenase-mediated digestion after 7 ± 2 days. Standard riboflavin/UVA crosslinking (Group B) approximately doubled tissue resistance, with complete degradation occurring after 14 ± 2 days. NAC/Polydatin treatment alone (Group C) produced a more modest increase in resistance (11 ± 2 days). The combination of NAC/Polydatin with CXL further prolonged tissue resistance. Pre-treatment before CXL (Group D) resulted in complete degradation after 17 ± 2 days, whereas post-treatment after CXL (Group E) prolonged resistance to 18 ± 2 days. The greatest effect was observed when NAC/Polydatin was administered both before and after CXL (Group F), with complete degradation occurring after 21 ± 2 days. One-way ANOVA demonstrated a significant overall difference among groups (F = 31.04, *p* = 3.01 × 10^−8^*)*. Tukey’s post hoc analysis confirmed significant differences for the comparisons highlighted in Figure 2, including untreated control versus CXL (*p* < 0.001), untreated control versus NAC/Polydatin pre/post-CXL (*p* < 0.001), and CXL alone versus NAC/Polydatin pre/post-CXL (*p* < 0.001). These findings demonstrate a progressive increase in resistance to collagenase-mediated degradation following CXL and a further enhancement when NAC/Polydatin is combined with CXL, with the strongest effect observed after pre- and post-CXL administration. Corneal degradation kinetics are illustrated in Figure 2 and Figure 3 and displayed in Table 1.

### 3.2. Scleral Degradation Time

Untreated scleral strips (Group A) underwent complete collagenase-mediated digestion after 10 ± 2 days (mean ± SD). Standard riboflavin/UVA crosslinking (Group B) markedly increased tissue resistance, prolonging complete degradation to 19 ± 3 days. NAC/Polydatin treatment alone (Group C) produced a more modest increase in resistance (14 ± 2 days).

The combination of NAC/Polydatin with CXL further enhanced scleral resistance to enzymatic degradation. Pre-treatment before CXL (Group D) resulted in complete degradation after 23 ± 2 days, whereas post-treatment after CXL (Group E) prolonged resistance to 24 ± 2 days. The greatest effect was observed in the NAC/Polydatin pre/post-CXL group (Group F), in which complete degradation occurred after 27 ± 3 days.

**Figure 4:**
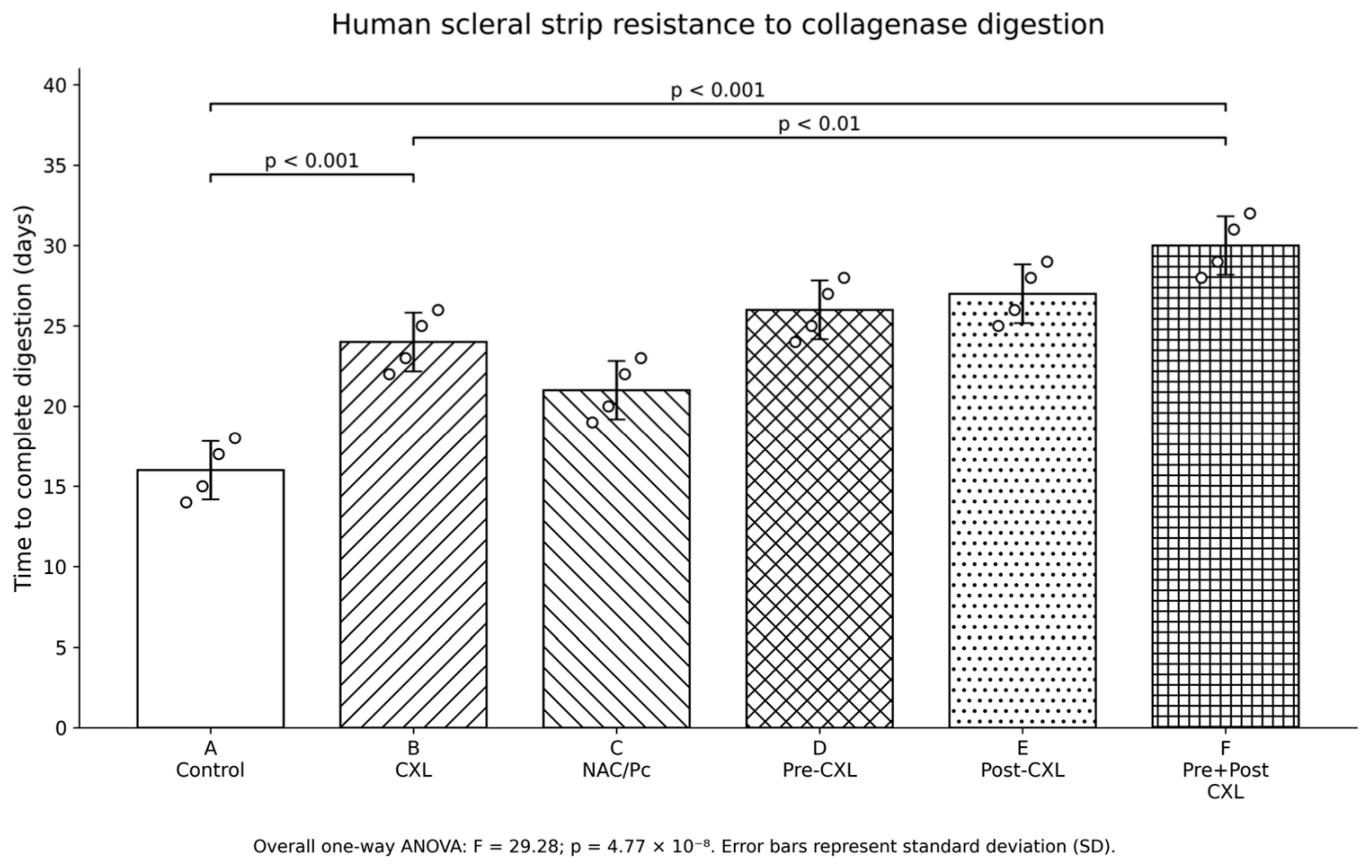
Resistance of human scleral strips to collagenase-mediated degradation following riboflavin/UVA crosslinking and adjunctive NAC/Polydatin treatment. Mean time to complete collagenase-mediated degradation of human scleral strips in the six experimental groups. Riboflavin/UVA collagen crosslinking significantly prolonged scleral preservation compared with untreated controls. NAC/Polydatin incubation alone produced a more modest increase in degradation time, whereas combined NAC/Polydatin treatment before and after CXL produced the greatest anti-degradative effect. Individual sample values are displayed, and error bars represent standard deviations (SDs). Statistical significance was assessed by one-way ANOVA followed by Tukey’s multiple-comparison post hoc test. The brackets indicate the statistically significant comparisons displayed in the figure: untreated control versus CXL (*p* < 0.001), CXL versus NAC/Polydatin pre/post-CXL (*p* < 0.01), and untreated control versus NAC/Polydatin pre/post-CXL (*p* < 0.001). CXL = collagen crosslinking; NAC = N-acetylcysteine; Pc = *Polygonum cuspidatum* (Polydatin) dry extract.

**Figure 5:**
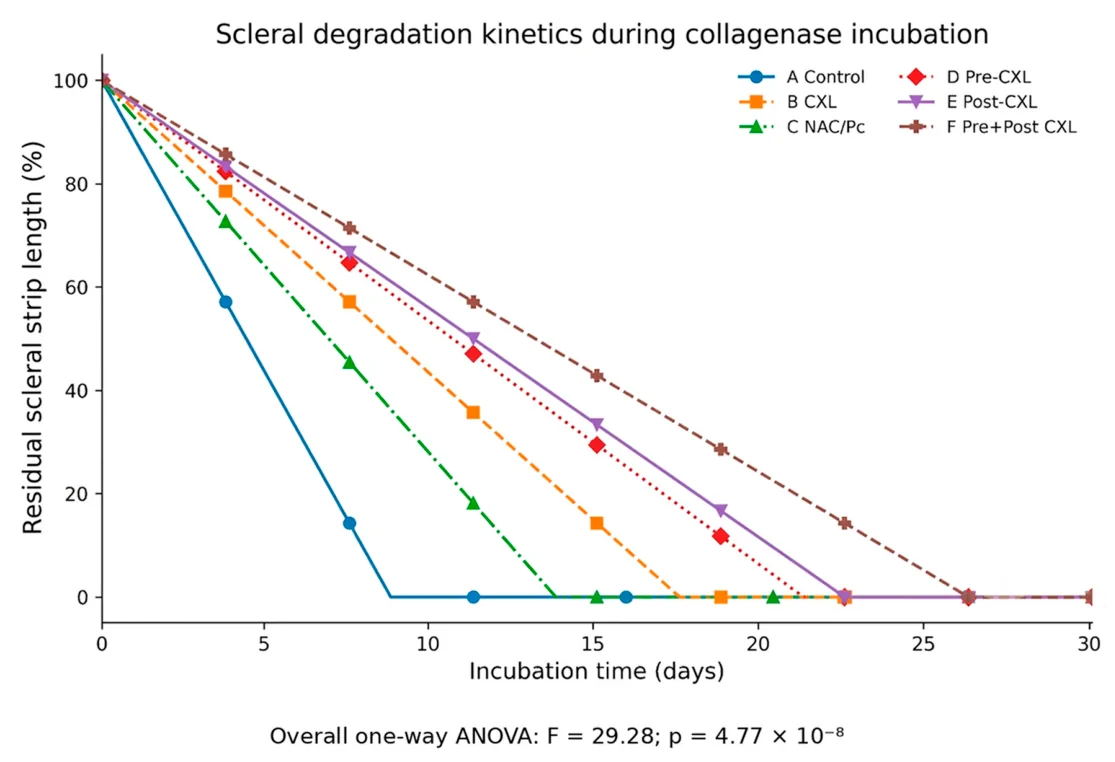
Kinetics of collagenase-mediated degradation of human scleral strips. Residual scleral length (%) was monitored over time in the six experimental groups. Untreated controls showed the fastest degradation, whereas riboflavin/UVA CXL delayed tissue digestion. The addition of NAC/Polydatin further enhanced tissue resistance, with the greatest preservation observed in the NAC/Polydatin pre/post-CXL group. Data are presented as means ± SDs. The overall statistical significance reported in the figure refers to the comparison of complete degradation times among the six experimental groups by one-way ANOVA (F = 29.28, *p* = 4.77 × 10^−8^), followed by Tukey’s multiple-comparison post hoc test; no point-by-point statistical comparisons were performed along the kinetic curves. CXL = collagen crosslinking; NAC = N-acetylcysteine; Pc = *Polygonum cuspidatum* (Polydatin) dry extract.

One-way ANOVA demonstrated a significant overall difference among groups (F = 29.28, *p* = 4.77 × 10^−8^). Tukey’s post hoc analysis confirmed significant differences for the comparisons highlighted in Figure 4, including untreated control versus CXL (*p* < 0.001), untreated control versus NAC/Polydatin pre/post-CXL (*p* < 0.001), and CXL alone versus NAC/Polydatin pre/post-CXL (*p* < 0.01). These findings demonstrate that CXL significantly increases scleral resistance to collagenase-mediated degradation and that adjunctive NAC/Polydatin treatment further enhances this effect, with the greatest resistance observed following combined pre- and post-CXL administration. Scleral degradation kinetics are shown in Figure 4 and Figure 5 and summarized in Table 1.

### 3.3. Protein Release Kinetics

**Figure 6:**
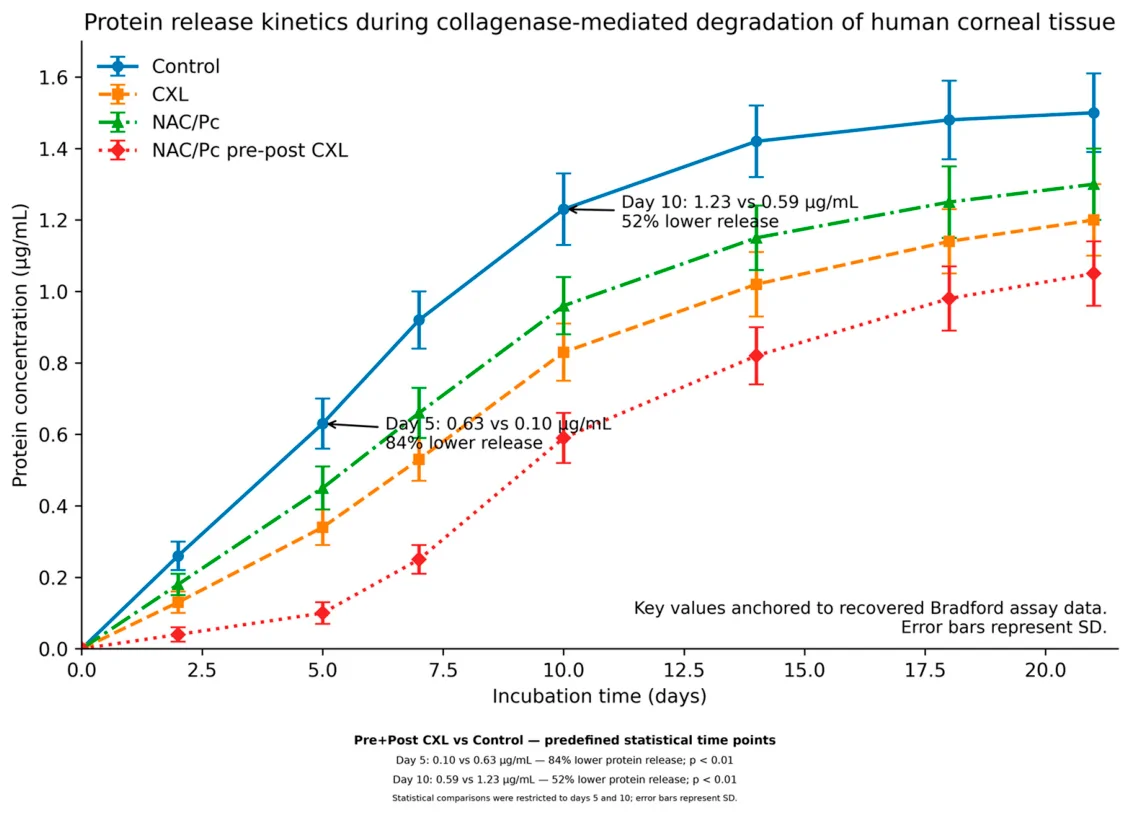
Protein release kinetics during collagenase-mediated degradation of human corneal tissue. Protein concentration in the supernatant was quantified by Bradford assay and is expressed as means ± SDs. Untreated controls showed the highest protein release, whereas CXL reduced protein release during collagenase-mediated degradation. The greatest reduction was observed in the NAC/Polydatin pre/post-CXL group. Statistical comparisons were restricted to the two predefined representative time points, days 5 and 10, and were performed between the untreated control and NAC/Polydatin pre/post-CXL groups using an unpaired *t*-test. At day 5, protein release was 84% lower in the NAC/Polydatin pre/post-CXL group than in untreated controls (0.10 vs. 0.63 µg/mL; *p* < 0.01). At day 10, protein release remained 52% lower (0.59 vs. 1.23 µg/mL; *p* < 0.01). Error bars represent standard deviations (SDs). CXL = collagen crosslinking; NAC = N-acetylcysteine; Pc = *Polygonum cuspidatum* (Polydatin) dry extract.

Protein release into the collagenase-containing supernatant increased progressively over time in all experimental groups, reflecting ongoing extracellular matrix degradation. Untreated control samples showed the highest protein release, whereas CXL reduced the amount of protein released during enzymatic digestion. NAC/Polydatin treatment further enhanced this protective effect, with the lowest protein concentrations consistently observed in the NAC/Polydatin pre/post-CXL group (Figure 6). Statistical comparisons were restricted to the two predefined representative time points, days 5 and 10. At day 5, the NAC/Polydatin pre/post-CXL group showed an 84% lower protein concentration compared with untreated controls (0.10 vs. 0.63 µg/mL; *p* < 0.01). At day 10, protein release remained 52% lower in the NAC/Polydatin pre/post-CXL group compared with controls (0.59 vs. 1.23 µg/mL; *p* < 0.01). These findings indicate that the combined pre- and post-CXL NAC/Polydatin treatment significantly reduced collagenase-mediated protein release at both representative time points analyzed. Overall, these results support the degradation-time findings and indicate that the combination of CXL with NAC/Polydatin provides greater resistance to enzymatic matrix degradation than CXL alone, with the strongest protective effect observed following pre- and post-CXL administration. Representative protein concentration profiles are shown in Figure 6.

## 4. Discussion

The present study demonstrated that riboflavin/UVA collagen crosslinking (CXL) significantly enhances resistance of human corneal and scleral tissues to collagenase-mediated enzymatic degradation. Furthermore, adjunctive treatment with N-acetylcysteine (NAC) and Polydatin further potentiated this anti-degradative effect when combined with CXL, whereas NAC/Polydatin incubation alone produced a more modest effect.

These findings support the concept that photochemical collagen stabilization represents a likely contributor to corneal and scleral tissue preservation, while adjunctive biochemical modulation may enhance extracellular matrix stabilization when associated with crosslinking treatment.

The anti-enzymatic effect observed after corneal crosslinking is consistent with the experimental findings previously reported by Spoerl et al. [5] and Aldahlawi et al. [27], who demonstrated increased resistance of crosslinked corneal tissue to pepsin, trypsin, and collagenase digestion [28]. The present findings further expand this anti-degradative paradigm by demonstrating similar effects in human scleral tissue and by exploring adjunctive extracellular matrix-protective strategies based on NAC and polydatin treatment.

Untreated corneal buttons underwent complete degradation after approximately 7 days, whereas crosslinked corneas remained structurally preserved for approximately 14 days, confirming a two-fold increase in resistance following riboflavin/UVA treatment. The protective effect induced by crosslinking is likely multifactorial. Riboflavin/UVA irradiation generates reactive oxygen species leading to additional intermolecular and intramolecular collagen bonds within the extracellular matrix [29,30]. These structural modifications increase tissue stiffness and reduce accessibility of collagen cleavage sites to proteolytic enzymes [31].

A major finding of the present study was the demonstration that adjunctive incubation with NAC [6] and Polydatin [10] further enhanced resistance to enzymatic degradation in both corneal and scleral samples. A protective effect was observed in samples receiving NAC/Pc treatment both before and after crosslinking, extending corneal degradation times up to approximately 21 days and scleral degradation times up to approximately 30 days.

N-acetylcysteine [7] is a well-known antioxidant and glutathione precursor with documented anti-inflammatory and anti-proteolytic properties [8]. Similarly, Polydatin has demonstrated antioxidant and extracellular matrix-protective effects in several experimental models [9]. Although the precise molecular mechanisms were not specifically investigated in the present study, the observed prolongation of tissue preservation suggests that adjunctive NAC/Polydatin treatment may complement the anti-degradative effects induced by riboflavin/UVA crosslinking. However, because mechanistic analyses were not performed, the underlying biological pathways remain hypothetical and warrant further investigation. The additional prolongation of tissue preservation observed after NAC/Polydatin exposure may reflect complementary anti-proteolytic and extracellular matrix-preserving actions, including previously reported modulation of matrix metalloproteinases, rather than a direct effect on the photochemical crosslinking process itself.

Although the clinical role of corneal crosslinking is now well established in Keratoconus [2,4] and iatrogenic post-laser vision correction corneal ectasia [30], supplemental extracellular matrix stabilization strategies may also have potential relevance in scleral remodeling associated with progressive myopia [11]. Progressive myopia is increasingly recognized as a disease characterized by collagen disorganization, protease activation, and biomechanical weakening of the scleral extracellular matrix, ultimately contributing to eye expansion and axial elongation [12,13].

In the present study, untreated scleral strips demonstrated greater intrinsic resistance to collagenase-mediated degradation compared with corneal tissue, likely reflecting the denser collagen architecture and lower hydration of scleral connective tissue [14]. Nevertheless, Riboflavin/UVA crosslinking significantly prolonged scleral preservation against enzymatic degradation, while adjunctive NAC/Polydatin exposure further enhanced resistance to extracellular matrix breakdown. These findings provide preliminary experimental evidence supporting the concept that combined biomechanical and biochemical stabilization strategies may reduce scleral ECM remodeling, thus enhancing its preservation.

The protein release kinetics further supported the morphologic findings. Untreated corneal samples demonstrated earlier and greater protein release into the incubation medium, whereas crosslinked and adjunctively treated tissues showed delayed protein release kinetics, suggesting slower extracellular matrix degradation and improved tissue preservation.

The collagenase-mediated digestion assay used in the present study should be interpreted as an accelerated ex vivo challenge model rather than as a direct simulation of physiological extracellular matrix turnover in vivo. The 0.1% collagenase A concentration and experimental conditions were deliberately selected to reproduce the established enzymatic degradation protocol originally described by Spoerl et al. [5], thereby providing a standardized and reproducible proteolytic challenge for comparing the relative resistance of differently treated tissues. Under these conditions, the purpose of collagenase exposure is not to reproduce the concentration, temporal profile, or complex regulation of collagenolytic enzymes occurring in the living cornea or sclera, but rather to generate a sufficiently controlled enzymatic stress to reveal treatment-dependent differences in extracellular matrix resistance.

Indeed, extracellular matrix remodeling in vivo is considerably more complex and involves the coordinated activity of multiple matrix metalloproteinases, endogenous tissue inhibitors of metalloproteinases, inflammatory mediators, resident cells, mechanical loading, and tissue-specific metabolic and repair mechanisms that are necessarily absent or markedly altered in an ex vivo model. Accordingly, the prolonged degradation times observed after CXL and adjunctive NAC/Polydatin treatment should not be interpreted as predicting the temporal behavior or rate of extracellular matrix degradation in vivo. Rather, they demonstrate increased relative resistance to a standardized collagenolytic challenge under controlled experimental conditions.

The use of this established accelerated model nevertheless offers an important experimental advantage: all tissue groups are exposed to the same defined enzymatic challenge, allowing the effects of CXL and adjunctive treatment on matrix resistance to be directly compared while minimizing biological and environmental variability. The present findings should therefore be regarded as proof-of-concept evidence of enhanced ex vivo resistance to enzymatic degradation, requiring confirmation in larger studies and in experimental models that more closely reproduce the cellular, biochemical, and biomechanical environment of the living cornea and sclera.

Several limitations of the present study should be acknowledged. First, the relatively small number of standardized biological specimens included in the quantitative analysis limits statistical power and generalizability. Although the complete experimental protocol was independently repeated in a second experimental session using tissues from different donors and reproduced the same overall pattern of treatment effects, the specimens available for the replicate experiment were smaller than those used in the primary experiment. The two datasets were therefore not pooled for quantitative statistical analysis in order to avoid introducing specimen-size heterogeneity as an additional source of variability. This independent replication provides supportive evidence for the reproducibility of the observed treatment pattern but does not substitute for validation in a larger, adequately powered study using standardized tissue dimensions and a greater number of independent human donor specimens. Second, the accelerated ex vivo collagenase digestion model cannot fully reproduce the complexity of extracellular matrix remodeling in vivo, including cellular responses, inflammatory pathways, mechanical loading, and tissue-specific repair mechanisms. Third, the precise contribution of direct collagenase inhibition versus tissue-level extracellular matrix stabilization induced by NAC and Polydatin was not specifically investigated. Moreover, the present study evaluated the combined NAC/Polydatin formulation, and therefore the individual contribution of each component cannot be determined. Future studies evaluating NAC and Polydatin separately, as well as different concentrations and exposure durations, will be required to characterize their respective effects and establish concentration–response and time–response relationships.

Despite these limitations, the present study provides novel evidence that combined Riboflavin/UVA crosslinking increase scleral digestion time and adjunctive NAC/Polydatin treatment may enhance resistance of human corneal and scleral tissues to collagenase-mediated degradation. These findings support further translational investigation of combined extracellular matrix stabilization strategies in corneal ectatic disorders and progressive myopia.

Interestingly, NAC/Polydatin incubation alone produced a more modest increase in tissue resistance, whereas its use in association with CXL, particularly in the pre/post-CXL protocol, further prolonged tissue preservation. This pattern suggests that the bioactive compounds may not independently induce substantial collagen stabilization but may potentiate the anti-degradative effect of photochemical crosslinking in an exposure-dependent manner. Future studies testing different concentrations and incubation times are required to confirm a true dose–response relationship.

## 5. Conclusions

Riboflavin/UVA collagen crosslinking significantly enhances the resistance of human corneal and scleral tissues to collagenase-mediated enzymatic degradation. Adjunctive NAC and Polydatin treatment may further enhance this anti-degradative effect, with the greatest tissue resistance observed following combined pre- and post-CXL administration. These findings provide preliminary proof-of-concept evidence, supporting further investigation of combined photochemical and biochemical extracellular matrix stabilization strategies in corneal and scleral tissues. However, given the ex vivo nature of the model and the limited sample size, further studies using larger cohorts, different NAC/Polydatin concentrations and exposure times, and experimental models more closely reproducing the in vivo environment are required before clinical extrapolation, particularly with regard to potential applications in progressive myopia.

## Figures and Tables

**Table 1:** Collagenase-mediated degradation times of human corneal buttons and scleral strips following riboflavin/UVA crosslinking and adjunctive NAC/Polydatin treatment. Data are expressed as means ± SDs.

Group	Treatment	Corneal Degradation Time (Days)	Scleral Degradation Time (Days)
A	Untreated control	7 ± 2	10 ± 2
B	Riboflavin/UVA CXL alone	14 ± 2	19 ± 3
C	NAC/Polydatin alone	11 ± 2	14 ± 2
D	NAC/Polydatin pre-CXL	17 ± 2	23 ± 2
E	NAC/Polydatin post-CXL	18 ± 2	24 ± 2
F	NAC/Polydatin pre- and post-CXL	21 ± 2	27 ± 3

CXL = collagen crosslinking; NAC = N-acetylcysteine; Polydatin = *Polygonum cuspidatum* dry extract.

## Data Availability

The data presented in this study are available on request from the corresponding author. The corneal tissues used in the study were provided by the Fondazione Banca degli Occhi del Veneto (Venice, Italy). These tissues were deemed unsuitable for transplantation and utilized in strict compliance with the ethical and regulatory guidelines governing the use of human tissues for scientific research purposes.

## Abbreviations

The following abbreviations are used in this manuscript:

KC	Keratoconus
UVA	Ultraviolet–A
Pc	*Poligonum cuspidatum*
NAC	N-acetylcysteine
CXL	Crosslinking

## References

[1] Wollensak G., Spoerl E., Seiler T. (2003). Riboflavin/ultraviolet-A–induced collagen crosslinking for the treatment of keratoconus. Am. J. Ophthalmol..

[2] Hafezi F., Kling S., Hafezi N.L., Aydemir M.E., Lu N.J., Hillen M., Knyazer B., Awwad S., Mazzotta C., Kollros L. (2025). Corneal cross-linking. Prog. Retin. Eye Res..

[3] Wollensak G., Spoerl E., Seiler T. (2003). Stress-strain measurements of human and porcine corneas after riboflavin–ultraviolet-A-induced cross-linking. J. Cataract. Refract Surg..

[4] Raiskup F., Herber R., Lenk J., Pillunat L.E., Spoerl E. (2024). Crosslinking with UV-A and riboflavin in progressive keratoconus: From laboratory to clinical practice—Developments over 25 years. Prog. Retin. Eye Res..

[5] Spoerl E., Wollensak G., Seiler T. (2004). Increased resistance of crosslinked cornea against enzymatic digestion. Curr. Eye Res..

[6] Ramaesh T., Ramaesh K., Riley S.C., West J.D., Dhillon B. (2012). Effects of N-acetylcysteine on matrix metalloproteinase-9 secretion and cell migration of human corneal epithelial cells. Eye Vis..

[7] Aldini G., Altomare A., Baron G., Vistoli G., Carini M., Borsani L., Sergio F. (2018). N-Acetylcysteine as an antioxidant and disulphide breaking agent: The reasons why. Free Radic. Res..

[8] Tenório M.C.D.S., Graciliano N.G., Moura F.A., Oliveira A.C.M., Goulart M.O.F. (2021). N-Acetylcysteine (NAC): Impacts on Human Health. Antioxidants.

[9] Du Q.H., Peng C., Zhang H. (2013). Polydatin: A review of pharmacology and pharmacokinetics. Pharm. Biol..

[10] Quinty V., Colas C., Nasreddine R., Nehmé R., Piot C., Draye M., Destandau E., Da Silva D., Chatel G. (2023). Screening and Evaluation of Dermo-Cosmetic Activities of the Invasive Plant Species Polygonum cuspidatum. Plants.

[11] Sun Y., Sha Y., Yang J., Fu H., Hou X., Li Z., Xie Y., Wang G. (2024). Collagen is crucial target protein for scleral remodeling and biomechanical change in myopia progression and control. Heliyon.

[12] McBrien N.A., Gentle A. (2003). Role of the sclera in the development and pathological complications of myopia. Prog. Retin. Eye Res..

[13] Rada J.A., Shelton S., Norton T.T. (2006). The sclera and myopia. Exp. Eye Res..

[14] Atchison D.A., Jones C.E., Schmid K.L., Pritchard N., Pope J.M., Strugnell W.E., Riley R.A. (2004). Eye shape in emmetropia and myopia. Investig. Ophthalmol. Vis. Sci..

[15] Holden B.A., Fricke T.R., Wilson D.A., Jong M., Naidoo K.S., Sankaridurg P., Wong T.Y., Naduvilath T.J., Resnikoff S. (2016). Global Prevalence of Myopia and High Myopia and Temporal Trends from 2000 through 2050. Ophthalmology.

[16] Wollensak G., Spoerl E. (2004). Collagen crosslinking of human and porcine sclera. J. Cataract. Refract Surg..

[17] Wollensak G., Iomdina E. (2009). Biomechanical and histological changes after collagen crosslinking of the sclera in rabbits. Acta Ophthalmol..

[18] Liu S., Li S., Wang B., Lin X., Wu Y., Liu H., Liu H., Qu X., Dai J., Zhou X. (2016). Scleral Cross-Linking Using Riboflavin UVA Irradiation for the Prevention of Myopia Progression in a Guinea Pig Model: Blocked Axial Extension and Altered Scleral Microstructure. PLoS ONE.

[19] Li Y., Qi Y., Sun M., Zhai C., Wei W., Zhang F. (2023). Clinical Feasibility and Safety of Scleral Collagen Cross-Linking by Riboflavin and Ultraviolet A in Pathological Myopia Blindness: A Pilot Study. Ophthalmol. Ther..

[20] Rong S., Wang C., Han B., Feng P., Lan W., Gao Z., Li X., Chen W. (2017). Iontophoresis-assisted accelerated riboflavin/ultraviolet A scleral cross-linking: A potential treatment for pathologic myopia. Exp. Eye Res..

[21] Gawargious B.A., Le A., Lesgart M., Ugradar S., Demer J.L. (2020). Differential Regional Stiffening of Sclera by Collagen Cross-linking. Curr. Eye Res..

[22] Huang Y., Gao R., Li Z., Chen A., Jiang Q., Ding S., Chen M., Meek K.M., Wang Q., Chen Z. (2025). The effects of different riboflavin concentrations and infiltration times on rabbit scleral crosslinking. Exp. Eye Res..

[23] Mazzotta C., Raiskup F., Hafezi F., Torres-Netto E.A., Armia Balamoun A., Giannaccare G., Bagaglia S.A. (2021). Long term results of accelerated 9 mW corneal crosslinking for early progressive keratoconus: The Siena Eye-Cross Study 2. Eye Vis..

[24] Burns F.R., Stack M.S., Gray R.D., Paterson C.A. (1989). Inhibition of purified collagenase from alka-li-burned rabbit corneas. Invest Ophthalmol. Vis. Sci..

[25] Kimmitt B.A., Moore G.E., Stiles J. (2018). Comparison of the efficacy of various concentrations and combinations of serum, ethylenediaminetetraacetic acid, tetracycline, doxycycline, minocycline, and N-acetylcysteine for inhibition of collagenase activity in an in vitro corneal degradation model. Am. J. Vet. Res..

[26] Bradford M.M. (1976). A rapid and sensitive method for the quantitation of microgram quantities of protein utilizing the principle of protein-dye binding. Anal. Biochem..

[27] Aldahlawi N.H., Hayes S., O’Brart D.P., Meek K.M. (2015). Standard versus accelerated riboflavin-ultraviolet corneal collagen crosslinking: Resistance against enzymatic digestion. J. Cataract. Refract Surg..

[28] Aldahlawi N.H., Hayes S., O’Brart D.P.S., O’Brart N.D., Meek K.M. (2016). An investigation into corneal enzymatic resistance following epithelium-off and epithelium-on corneal cross-linking protocols. Exp. Eye Res..

[29] Weadock K.S., Miller E.J., Keuffel E., Dunn M.G. (1996). Effect of physical crosslinking methods on collagen-fiber durability in proteolytic solutions. J. BioMed Mater. Res..

[30] Weadock K.S., Miller E.J., Bellincampi L.D., Zawadsky J.P., Dunn M.G. (1995). Physical crosslinking of collagen fibers: Comparison of ultraviolet irradiation and dehydrothermal treatment. J. BioMed Mater. Res..

[31] Mallya S.K., Mookhtiar K.A., Van Wart H.E. (1992). Kinetics of hydrolysis of type I, II, and III collagens by the class I and II *Clostridium histolyticum* collagenases. J. Protein Chem..

